# The National Medical Union

**Published:** 1914-02-07

**Authors:** Robert Carswell, E. Arthur Dorrell

**Affiliations:** Hon. Secretary; Hon. Secretary


### THE PROFESSION OF MEDICINE.

### The National Medical Union.

A meeting of the Federating Council was held at the offices of the Union, 346 Strand, on Saturday, January 31. It was announced at the beginning of the meeting that the Islington Non-Panel Association had decided to join the Federation, and that at a meeting of non-panel practitioners in Bradford it was resolved to form a local branch in that neighbourhood.

The report of the Executive Committee was submitted to the meeting by Dr. Greenyer, who stated that the Executive Committee had met on December 20, 1913, and on January 17, 1914. He announced that it had engaged and furnished an office at 346 Strand, that it had secured clerical assistance, and that it had discussed and issued to the press a short general memorandum of policy for the purpose of making known the existence of the Union. The remainder of the report was embodied in recommendations to the Council which were moved by the Chairman.

(*a*) It was resolved that an Organisation Committee be formed, consisting of the members of the Executive Committee, with powers of co-option and of obtaining co-operation of suitable local members, for the purpose of enrolling members in the Union; its existence being determinable on the date of the first general meeting of the Union. The Organisation Committee will meet for the first time on Saturday, February 7, at the offices of the Union, at 4.30 p.m.

(*b*) It was resolved that a Constitution Committee be appointed to consider the most suitable form of constitution for the purposes of the Union, and to draft proposals thereon, the opinion of the Council being that any questions pertaining to trade unionism need not be considered.

(*c*) After considerable discussion of a resolution providing that no member of the Union shall sign Form 43 IC it was resolved that this question be referred to a full general meeting of the Union, with an expression of opinion from the Council that no member shall be allowed to sign this form or any similar form in relation to any patient for whom he has not already signed it.

(*d*) It was resolved that practitioners attending members of the Seamen's National Insurance Society shall not be debarred from belonging to the National Medical Union. But the Council of the National Medical Union recommend practitioners not to sign the agreement of the Seamen's National Insurance Society.

(*e*) It was resolved to accept Dr. Heatherley, of Liverpool, as a member of the Executive Committee in place of Dr. O'Sullivan.

Dr. Prowse, on the instruction of the Manchester Union, moved the following recommendations:—

(*a*) That the cases of those members of the medical profession whose partners are upon the panel, but who do not see their partners' patients, and whose deeds of partnership were in existence before the panels were formed, be considered.

Whereupon it was resolved that such practitioners should not thereby be excluded from membership of the National Medical Union, if approved by the Council of the Union, after recommendation by their local non-panel association.

(*b*) That the question of policy should be considered at once, and that in this connection the system of administration of medical benefit adopted by the National Deposit Friendly Society and the Scottish Clerks' Association should be gone into very carefully.

It was resolved that this recommendation be entered upon the minutes.

(*c*) That the Council consider the advisability of enlarging the power of a member of Council to name a delegate to represent him at a meeting which he is unable to attend personally.

It was resolved that it was *ultra vires* for the Council to deal with this matter, and it was referred back to be dealt with at a general meeting of the Union.

(*d*) That a federating association should be permitted to adopt the title of "Branch" of the National Medical Union, if such is the desire of its members.

It was resolved that the word "Branch" was not permissible, but such title as the "National Medical Union, Manchester District," was very suitable.

(*e*) That the time has arrived for the formation of a definite policy with regard to taking steps for the amendment of the existing National Insurance Acts, or for the drafting of a new Act more in consonance with the views of the medical profession.

Whereupon it was resolved that the secretaries be instructed to reply that the local associations are requested, as soon as they have sufficiently pushed enrolment of members, to formulate suggestions for a policy to be forwarded to the Federating Council.

(*f*) That the Council at once take legal advice as to the validity of the medical certificates issued by non-panel medical men; and that the effect of the decisions given in the Courts of Justice be ascertained and published to all members, and in the public press.

It was considered that there was no doubt of the validity of the judgment in the case of Heard *v.* Pickthorne, and it was agreed that the latter part of the recommendation should be noted in view of taking action.

A written copy of Regulation 44 (2) was laid before the meeting by Dr. Greene. This Regulation reads as follows:

"Where the insured person contracts with a person other than a duly qualified practitioner to obtain treatment (whether including drugs and appliances or not) from him for a fixed sum for the year, or any part thereof, the Committee may make such contribution towards the sum contracted to be paid not exceeding in amount the maximum contribution payable in the case of a person who contracts with a duly qualified medical practitioner, as they think fit, but upon any representation being made by a society that the treatment is not such as will adequately protect the funds of the society, the Committee may either withhold the contribution, or make such a deduction therefrom as they may in any case determine."

Whereupon it was resolved that the Council protests against the Regulation 44 (2) of the National Insurance Commissioners, containing provisions which seek to legalise the treatment of those insured under the Act by persons who are not duly qualified practitioners, and instructs the secretaries to bring the terms of the Regulation to the notice of the General Medical Council and to request them to take action.

Robert Carswell, }

E. Arthur Dorrell, } Hon. Secretaries.

